# Cancer awareness in the State of Kerala in India

**DOI:** 10.3332/ecancer.2025.1923

**Published:** 2025-06-05

**Authors:** Association of Medical and Pediatric Oncologists of Kerala (AMPOK)

**Affiliations:** Ernakulam Medical Centre and MOSC Medical College, Ernakulam 682028, Kerala, India

**Keywords:** cancer awareness, Kerala, cancer knowledge, cancer risk factors, cancer causes, cancer control, India

## Abstract

Kerala is experiencing a rising cancer burden driven by demographic transitions and unhealthy lifestyle patterns. Recognising the urgent need to address these challenges, the Association of Medical and Pediatric Oncologists of Kerala conducted a comprehensive survey to evaluate public awareness regarding cancer risk factors, prevention strategies and screening practices. The survey, conducted between October and December 2024, employed a mixed-method approach using online and door-to-door data collection to ensure inclusivity across diverse demographic groups. The findings revealed a high general awareness of cancer symptoms and risk factors. However, significant gaps persisted in knowledge about preventive measures such as vaccination and genetic testing. Despite heightened awareness, screening rates remained low. The survey findings underscore the importance of tailored educational interventions to address misconceptions, promote preventive behaviours and enhance access to affordable screening and treatment services. Lessons from this study hold relevance for global oncology programs, offering opportunities for bidirectional learning to enhance cancer awareness and prevention initiatives worldwide.

## Introduction

Kerala, a southern state in India, is widely recognised for its high human development index and impressive health indicators. However, like many regions undergoing epidemiological transitions, Kerala is witnessing a rise in the burden of non-communicable diseases, particularly cancer [1]. This alarming trend is attributed primarily to demographic shifts, particularly an aging population, coupled with the increasing prevalence of unhealthy lifestyles [2] Behavioural and environmental factors such as tobacco consumption, alcohol use, poor dietary habits, physical inactivity and rising obesity rates have emerged as significant contributors to the escalating incidence of cancer in the state [3]. Understanding the role of these determinants is critical for implementing effective preventive and control measures.

The state government has established a State Cancer Control Board and adopted the Kerala Cancer Control Strategy 2018–2030, emphasising a decentralised approach to cancer control and care [4] This strategy aims to enhance access to early diagnosis, strengthen preventive services at the primary care level and ensure convenient access to cancer treatment. However, achieving equitable implementation across the state remains a key challenge in effectively reducing Kerala’s cancer burden. The Association of Medical and Pediatric Oncologists of Kerala (AMPOK) is a professional society representing cancer care specialists dedicated to improving outcomes for patients across the state. AMPOK members collectively care for lakhs of individuals with cancer, addressing the diverse challenges associated with diagnosis, treatment and survivorship. Recognising the growing cancer burden and the need for effective interventions, the AMPOK society hypothesised that there are gaps in awareness regarding cancer risk factors and prevention strategies among the general population. Addressing these gaps is essential for promoting early detection and preventive practices, thereby reducing cancer-related morbidity and mortality.

Considering these concerns, AMPOK conducted a comprehensive study to evaluate public awareness of cancer, its associated risk factors and preventive strategies among residents of Kerala. The primary objective was to generate evidence that could guide health education campaigns and inform policy decisions aimed at cancer prevention and early detection. This report presents the findings of the study, highlighting key insights into the population’s knowledge, attitudes and practices related to cancer prevention. It also outlines recommendations for strengthening public health initiatives to mitigate the cancer burden in Kerala.

## Methods

A team of oncologists from the AMPOK formulated a questionnaire to study some of the key questions that can inform policymaking on cancer control and care. The survey questionnaire was meticulously designed to assess common perceptions and misconceptions about cancer, evaluate knowledge of risk factors and gauge attitudes toward screening and prevention practices. Additionally, it incorporated items to explore barriers to cancer prevention and early detection. A pilot testing was conducted among a group of doctors who are not cancer specialists. We sought feedback on the language of the questionnaire and refined the questions to reflect this feedback. The questionnaire was disseminated in the Malayalam language, which is spoken by more than 99% of the population in the state. We surveyed persons living in Kerala aged 18 and above with both a convenience and random sampling strategy. We utilised online form-based surveys to reach individuals with smartphone access and print-based surveys to engage those with limited internet access. We did not use the IP address-based blocking strategy for preventing duplicate responses. This dual strategy ensured inclusivity and broader participation across various demographic groups, including rural and urban populations. The survey was initially distributed through residents’ association WhatsApp groups, which then snowballed into other Kerala-based networks. To improve coverage in rural areas, we supplemented this with printed surveys delivered door-to-door. This approach continued until we achieved a response pool that adequately reflected the state’s demographic distribution. We did not provide correct answers to the questions on the survey or counseling to the participants. The state of Kerala has good coverage of primary healthcare facilities and, therefore, participants have access to nurses or doctors in nearby locations should they have concerns following the survey participation.

Given the estimated adult population size of 33.4 million in Kerala, we determined a minimum required sample size of 385 across both rural and urban participants to achieve 95% confidence with a 5% margin of error for 50% response distribution. However, considering potential non-responses and to ensure robust data collection, we targeted a larger sample size of at least 1,000 adults. This approach was aimed at improving the statistical power and representativeness of the findings.

A questionnaire was designed to investigate the awareness of the participants on various cancer-related topics. Data collection was facilitated by trained survey administrators and community volunteers to enhance response rates and ensure accurate data recording.

The survey was conducted over a 2-month period from October to December 2024. During this time, concerted efforts were made to engage diverse segments of the population, including those from various socioeconomic and educational backgrounds. Confidentiality and anonymity were maintained throughout the process. Data was collected using an online form as well as the door-to-door method, especially in rural areas of the state. Multiple choice questions were used for the study. The collected data were subsequently analysed using descriptive statistical methods to identify patterns and draw meaningful conclusions regarding cancer awareness and prevention behaviours among the respondents.

## Results

The survey was completed by 2,443 individuals, of whom 2,361 were residing in Kerala. The online survey received 4,773 visits, out of whom 1,196 completed the survey – resulting in a response rate of approximately 25%. 50% of the survey participants used the online form for reporting their awareness, while the rest (1,165 responses) were obtained through door-to-door visits. Analysis was conducted on 2,361 responses. Table 1 provides an overview of the demographic characteristics of the study population. A significant proportion (60%) of respondents were between the ages of 31–65 years. Female respondents outnumbered males, accounting for 63% of the sample compared to 37% of male participants. Only 3% of respondents reported having no formal education, while more than 60% had pursued education beyond secondary school, including college, postgraduate or professional degrees. Rural residents constituted nearly 60% of the surveyed population, ensuring representation across geographic strata.

Awareness regarding cancer symptoms was high, with 90% of respondents claiming some level of awareness about common symptoms. Approximately 80% reported confidence in identifying early signs of cancer. Furthermore, 84% expressed concern about their personal risk of developing cancer during their lifetime, indicating a heightened perception of vulnerability Table 2 summarises the results of the survey study.

In terms of risk factor awareness, 57% of respondents confidently identified unhealthy lifestyle habits, such as poor dietary patterns, lack of physical activity and substance abuse, as contributors to cancer risk. An additional 36% believed lifestyle choices might influence cancer risk, while only 3% dismissed any connection between lifestyle and cancer. Specific knowledge about carcinogens was encouraging, with 87% recognising smoking as a causative factor, followed by 73% acknowledging the role of alcohol consumption. Dietary habits and hereditary factors were identified by 62%–52%, respectively. However, 18% incorrectly associated mobile phone use with cancer and 25% mistakenly linked safe pesticide use, as defined by regulatory guidelines, to cancer risk.

Concerning tobacco use, 85% of participants believed that over 50% of cancers in men could be attributed to tobacco consumption. A majority (91%) acknowledged a moderate to strong link between tobacco or alcohol use and cancer risk. Additionally, 61% felt that portrayals of tobacco use by celebrities influenced younger generations to adopt these habits.

Despite widespread awareness, cancer screening rates remained low, with 80% of respondents reporting that they had never undergone screening for cancer.

Regarding cancer care, 73% of respondents cited financial costs as a primary concern, while 38% were apprehensive about the effectiveness of cancer therapies. Nearly 50% expressed concerns about treatment-related side effects. Only 3% endorsed alternative therapies as sufficient for cancer management. While 41% favoured modern medical treatments exclusively, 38% supported a combined approach involving modern and AYUSH (Ayurveda, Unani, Siddha, Homeopathy and Naturopathy) therapies.

Knowledge about cancer prevention strategies was limited. Only 15% believed that vaccines could prevent certain types of cancer, and just 29% were aware of the role of genetic testing in prevention and early detection.

Perceptions about cancer prognosis were more optimistic, with only 17% viewing a cancer diagnosis as invariably fatal. Encouragingly, 66% of respondents believed there was no stigma associated with cancer within their communities.

Regarding sources of information, 70% of participants reported obtaining cancer-related knowledge from the internet and social media, while an equal proportion cited healthcare professionals as sources of information. Community health programs served as an additional source for 35% of respondents.

## Discussion

The AMPOK survey provided significant insights into cancer awareness and perceptions among the population in Kerala. Key findings include: Participants demonstrated a relatively high level of awareness regarding cancer symptoms and common risk factors. Tobacco use, alcohol consumption and unhealthy lifestyles were appropriately identified as contributors to cancer incidence. A noteworthy concern was expressed regarding the influence of sportspersons and cinema actors in promoting tobacco use among the youth, highlighting the perceived impact of cultural icons on health behaviours. Only a minority of participants regarded all cancers as invariably fatal, indicating a more nuanced understanding of cancer prognosis within the community.

Despite these positive findings, the survey also uncovered several gaps and opportunities for enhancing cancer control strategies in Kerala. Most participants expressed apprehension about the financial burden associated with cancer treatment, underscoring the need for more accessible and affordable care options. Few participants demonstrated awareness of cancer prevention strategies such as human papilloma virus vaccination to prevent cervical cancer, pointing to the need for targeted educational interventions to promote prevention-focused behaviours. Awareness regarding the role of genetic testing in cancer prevention and early diagnosis was found to be low, highlighting an area for further educational outreach.

A significant proportion of respondents endorsed the integration of modern medicine with unproven alternative therapies, reflecting the need for evidence-based communication strategies to address misconceptions. Despite high levels of general awareness, several myths persisted, such as the belief that mobile phone use can cause cancer and that any pesticide used in food production is harmful, even at safe levels. These misconceptions require targeted myth-busting campaigns. A substantial proportion of participants reported stigma related to cancer diagnosis and treatment, indicating the necessity of stigma-reduction interventions. Cancer screening rates were reported to be alarmingly low, emphasising the need for initiatives to promote routine screening and early detection practices. Currently, there is no systematic cancer screening program in Kerala. There are some government efforts to initiate state-wide awareness programs and screening camps focused on breast cancer.

The findings of the AMPOK survey highlight both strengths and deficiencies in cancer awareness and perceptions in Kerala. Addressing the identified gaps through comprehensive educational programs, policy interventions and community engagement can significantly improve cancer prevention, early detection and treatment outcomes in the region.

This survey-based study has inherent limitations. All survey studies are susceptible to sampling bias. However, the use of both online snowballing strategies for survey recruitment and door-to-door surveying was intended to mitigate this bias. The survey participants may not accurately represent the demographic composition of Kerala, particularly as older adults may be underrepresented and women are overrepresented. Nevertheless, the findings provide insights that are relevant to the broader community. While the results may not be generalisable to other states in India or internationally due to differences in educational and socioeconomic backgrounds, these variations are critical for identifying effective interventions. Additionally, another key limitation of the survey is that we did not define some terminologies – such as ‘safe pesticide use’ and ‘genetic testing for cancer prevention’. We believe these questions require further focused studies to assess the awareness on various aspects of these themes among the public.

Lessons learned from the AMPOK survey may have broader relevance to other regions globally experiencing a rising cancer burden. Comparative studies across diverse socioeconomic and educational contexts can inform strategies to improve cancer control efforts. The findings also provide an opportunity for contextual comparison with the American Society of Clinical Oncology (ASCO) survey conducted in 2020 (Table 3) [5]. Both surveys highlight the need for enhanced cancer education, though differences in awareness, attitudes and screening behaviours underscore the importance of region-specific approaches.

Awareness of cancer risk was higher among AMPOK respondents, with 84% expressing concern about their lifetime risk of cancer compared to 54% in the ASCO survey. This heightened concern in Kerala may reflect a stronger perceived vulnerability, possibly influenced by higher literacy levels and local awareness campaigns. Similarly, 93% of AMPOK respondents identified lifestyle factors, such as poor diet and physical inactivity, as contributors to cancer risk, significantly higher than the 34% reported in the ASCO survey, which focused on smoking, sun exposure and diet.

Knowledge of hereditary factors also differed. In the AMPOK survey, 52% of respondents recognised heredity as a cancer risk factor, while 28% in the ASCO survey wrongly believed most cancers were hereditary, and 66% acknowledged heredity as a risk factor. This highlights a need for clearer communication about genetic risks and the role of genetic testing in both populations.

Regarding specific carcinogens, 91% of AMPOK respondents associated tobacco and alcohol with cancer risk, compared to 80% in the ASCO survey identifying smoking as a risk factor and 34% linking alcohol consumption to cancer. In the ASCO survey, 10% of respondents identified environmental pollution as a cause of cancer, whereas in the AMPOK survey, 34% recognised it as a significant risk factor. Misconceptions were also observed; 18% of AMPOK participants associated mobile phone use with cancer compared to 14% in the ASCO survey, reflecting persistent myths that need to be addressed through targeted education.

In the AMPOK survey, 48% of respondents identified trans fats as a risk factor for cancer. In comparison, around 25% of respondents in the ASCO survey reported processed meats and artificial sweeteners as risk factors for cancer. Greater emphasis on communicating the risk of processed and ultra-processed food for causing cancer is essential in both communities.

When considering treatment preferences, only 3% of AMPOK respondents endorsed alternative therapies as the sole treatment modality for cancer care, compared to 35% in the ASCO survey. Additionally, 41% of AMPOK respondents preferred modern medicine exclusively, while 38% supported a combination of modern and AYUSH therapies. In contrast, 73% of ASCO respondents preferred integrating alternative and modern treatments, indicating cultural and systemic differences in healthcare approaches.

Education levels also revealed differences, with over 60% of AMPOK respondents having education beyond secondary school, compared to 34% in the ASCO survey. This disparity may partly explain the higher awareness levels observed in Kerala and highlights the potential for leveraging education to further improve awareness and preventive behaviours.

Most participants reported obtaining cancer-related awareness from both social media and online sources as well as healthcare professionals – underscoring the complementary roles of digital platforms and healthcare providers in disseminating cancer awareness.

In conclusion, the AMPOK survey demonstrates higher awareness of cancer risk factors and prevention strategies compared to the ASCO survey, but significant gaps remain in translating awareness into preventive actions, such as screening and cancer prevention. The findings underscore the need for context-specific interventions, including education campaigns to address misconceptions, policies to improve access to affordable screening and integration of genetic counseling services.

Future efforts should focus on leveraging Kerala’s high literacy and digital penetration to disseminate accurate information while enhancing infrastructure to support early detection and effective cancer care. The survey also showcases the poor state of cancer-related awareness in the United States. The AMPOK experience in enhancing cancer awareness in the state of Kerala can help in building resources for greater public health education in the United States. Such bidirectional learning in global oncology can help the more advanced economies in their cancer control programs.

## Conflicts of interest

No conflict of interests by the authors or the AMPOK society.

## Funding

None.

## Author contributions

Anju Anna Abraham, Favaz Ali M, Nidhun V Ashok, Manuparasad Avaronnan, Arun Chandrasekharan, Sarah Easaw, Jyothis P Jose, Mathews Jose, Aswin Joy, Ashok Sebastian Komaranchath, Sajeevan Kunivayalil, Suneesh Soman Nair, Prashant Parameswaran, Keechilat Pavithran, Arun Philip, Sanudev Sadanandan, Nithin Raj D, Varun Rajan, Sourabh Radhakrishnan, Azgar Abdul Rasheed, Sameer Salahuddin, Nikhil Sebastian, Sugeeth M Thambi, Boben Thomas, Antony George Francis ThottianDilip Harindran Vallathol, Narayanankutty Warrier, Arun V, Roja Joseph, Aju Mathew, Muneer A, Nikhil Krishna Haridas, M. Ponraj, Shoufeej Pulikkal Mukkath and Saikumar S.

## Figures and Tables

**Table 1.** Baseline characteristics of the survey population.

Characteristic	*N*	Percentage
Total respondents	2,443	100
Age group (Years)		
18–30	716	29
31–50	860	35
51–65	588	24
65-above	279	11
Gender		
Female	1,534	63
Male	909	37
Education level		
No formal education	82	3
Primary education	341	14
Secondary education	549	22
Undergraduate degree	718	29
Postgraduate degree	355	15
Professional	398	16
Residence		
Corporation	424	17
Municipality	485	20
Panchayat	1,452	60
Outside Kerala	82	3

**Table 2.** Results of the survey.

	*N*	Percentage
Total number of people residing in Kerala	2,361	100
How aware are you of the common symptoms of cancer?		
Very aware	369	16
Somewhat aware	1,747	74
Not aware at all	245	10
Do you believe that lifestyle play a role in cancer risk? (lifestyle – habits, diet, physical activity)		
Yes, definitely	1,336	57
Possibly	846	36
No, they do not	72	3
Unsure	107	5
What proportion of cancers in men in Kerala can be attributed to tobacco use?		
>75	896	38
50–75	1,118	47
25–50	285	12
<25	62	3
How strong is the association of tobacco and alcohol use with cancer?		
High	1,589	67
Moderate	564	24
Mild	137	6
Not associated with cancer	13	1
Unsure	58	2
Do you engage in regular check-ups or screening against cancer?		
Yes	460	20
No	1,901	80
What is your primary concern about cancer treatment? (Select all that apply)		
Side effects	1,137	48
Cost of treatment	1,714	73
Access and availability of treatment	445	19
Effectiveness	904	38
No concern	111	5
Unsure	87	4
Would you prefer a combination of modern medicine and alternative medicine (AYUSH -Ayurveda, Unani, Siddha, Homeopathy) for cancer treatment?		
Yes, both are important	901	38
Only modern treatments	972	41
Only alternative treatments	69	3
Unsure	419	18
Do you think any type of cancer can be prevented by taking a vaccine?		
Yes	349	15
No	1,352	57
Unsure	660	28
Which of the following do you believe are causes of cancer? (Select all that apply).		
Smoking	2,050	87
Alcohol consumption	1,725	73
Bad diet	1,465	62
Hereditary	1,238	52
Environmental pollution	812	34
Stress	353	15
Viral or bacterial infection	353	15
Radiation exposure	936	40
Mobile phone use	436	18
High voltage electric line	157	7
Save pesticide used for agriculture	588	25
Trans Fat in packet food	1,144	48
Do you believe that cancer is always fatal?		
Yes	391	17
No	705	30
It depends on the type of the cancer and stage of the cancer	1,150	49
Unsure	115	5
Where do you usually get information about cancer prevention and treatment? (Select all that apply)		
Healthcare professional	1,654	70
Internet and social media	1,648	70
TV and radio	706	30
Friends or family	715	30
Community health program	836	35
How confident are you in identifying early symptoms of cancer?		
Very confident	386	16
Somewhat confident	1,515	64
Not confident at all	460	20
How concerned are you about the possibility of developing cancer in your lifetime?		
Very concerned	600	25
Somewhat concerned	1,381	59
Not concerned	380	16
Do you think there is a stigma associated with a cancer diagnosis in your community?		
Yes	460	20
nNo	1,559	66
uUnsure	342	14
Do you think genetic testing can help in the prevention or early detection of cancer?		
Yes, definitely	679	29
Maybe	1,063	45
No, it's not effective	248	10
Unsure	371	16
Do you think tobacco used by actors and sportsmen negatively influences tobacco use among youngsters?		
Yes	1,431	61
No	656	28
Unsure	274	11

**Table 3.** ASCO Survey 2020 (USA) N = 4012 (includes 162 patients with cancer).

Age	Percentage
18–34	31
35–49	23
50–64	25
>65	21
Gender	
Male	47
Female	53
Education level	
Less than high school	9
High school to less than 4 years degree	57
4 years college degree or more	34
Residency	
Urban	33
Suburban	46
Rural	21
Which of the following do you think most cancers are caused by?	
Lifestyle choices (e.g., smoking, sun exposure, diet)	34
Family history (i.e., hereditary factors )	28
Environmental causes (e.g., pollutants)	10
Proportion of survey participants reporting risk factors for cancer (Which of the following do you think increases a person’s risk of getting cancer? Please select all that apply.)	
Family history/hereditary factors	66
Smoking e-cigarettes	53
Smoking cigarettes	80
Alcohol	34
Certain viral infections	22
Cellphone use	14
Processed meats	26
Artificial sweeteners	23
Use of other tobacco products (e.g., cigars, pipes, chewing tobacco, etc.)	63
Proportion of survey participants reporting alternative therapies as cancer treatment	
Cancer can be cured solely through alternative therapies, without standard cancer treatment.	35
Alternative therapies are a good supplement to standard cancer treatment	73
Proportion of survey participants reporting concerns about developing cancer in their lifetime	
Very concerned/somewhat concerned	54
Not at all/Not very concerned	41
Already diagnosed	5
Proportion of survey participants reporting concerns of being diagnosed with Cancer (What are/were your greatest concerns about being diagnosed with cancer? Please select all that apply).	
Side effects of the treatment	49
Paying for the treatment	42
